# Autistic traits predict obsessive-compulsive symptoms: Study in a clinical sample

**DOI:** 10.1192/j.eurpsy.2021.1115

**Published:** 2021-08-13

**Authors:** C. Pinto-Gouveia, A. Araujo, A.T. Pereira, C. Cabaços, S. Renca, A. Macedo

**Affiliations:** 1 Psychiatry Department, Centro Hospitalar e Universitário de Coimbra, Coimbra, Portugal; 2 Department Of Psychological Medicine, Faculty of Medicine, University of Coimbra, Coimbra, Portugal; 3 Institute Of Psychological Medicine, Faculty Of Medicine, University of Coimbra, coimbra, Portugal; 4 Institute Of Psychological Medicine, Faculty Of Medicine, University of Coimbra, Coimbra, Portugal

**Keywords:** neurocognitive processes, Obsessive-Compulsive disorder, autism spectrum disorder

## Abstract

**Introduction:**

Co-occurrence of obsessive-compulsive disorder (OCD) and autism spectrum disorder (ASD) features is well stablished. Diagnosis of OCD increases the risk of a later diagnosis of ASD, and vice versa. Moreover, a recent combined genome-wide association study identified a shared polygenic risk between the two disorders. Our preliminary results also indicate that OCD patients have higher levels of autistic traits than individuals from the community.

**Objectives:**

To determine which autistic dimensions (social skill, communication, attention switching, attention to detail imagination) are predictors of OC symptoms.

**Methods:**

39 OCD patients (52,5% female; 19 to 64 years old) answered the Portuguese versions of the Autism-Spectrum Quotient for Adults and Obsessive Compulsive Inventory – Revised (OCI-R). Spearman correlation and linear multiple regression tests were performed using SPSS.

**Results:**

The OCI-R global score showed positive correlations with some AQ dimensions (attention switching, attention to detail and communication). The regression model showed that attention to detail (β = .43, p = .01) and attention switching (β = .33, p = .038) explained 36% of obsessive-compulsive symptoms variance.

**Conclusions:**

Our results are in line with a dimensional perspective of psychopathological continua and indicate that the overlap between OCD and ASD occurs through shared neurocognitive processes. We suggest that, besides being a predisposing factor for social difficulties (e.g.: facial/emotion recognition) in ASD, attention to detail and deficits in attention switching may also lead to difficulties to dismiss repetitive thoughts or extinguish behaviours in OCD. Future studies should investigate the distinctive features and underlying processes between OCD/ASD.

